# Poly I:C induces collective migration of HaCaT keratinocytes via IL-8

**DOI:** 10.1186/s12865-017-0202-3

**Published:** 2017-04-24

**Authors:** Kazuhide Takada, Shihoko Komine-Aizawa, Naoko Hirohata, Quang Duy Trinh, Atsuyoshi Nishina, Hirokazu Kimura, Satoshi Hayakawa

**Affiliations:** 1 Division of Microbiology, Department of Pathology and Microbiology, Nihon University School of Medicine, 30-1 Oyaguchi Kami-cho, Itabashi-ku, Tokyo, 173-8610 Japan; 2 Division of Oral Surgery, Department of Otolaryngology-Head and Neck Surgery, Nihon University School of Medicine, 30-1 Oyaguchi Kami-cho, Itabashi-ku, Tokyo, 173-8610 Japan; 3 Department of Materials and Applied Chemistry, College of Science and Technology, Nihon University, 1-8-14, Kanda surugadai, Chiyoda-ku, Tokyo, 101-8308 Japan; 4 Infectious Disease Surveillance Center, National Institute of Infectious Diseases, Musashimurayama-shi, Tokyo 208-0011 Japan

**Keywords:** Collective migration, Epithelial-mesenchymal transition, IL-8, Keratinocyte, Poly I:C, Toll-like receptor, Wound healing

## Abstract

**Background:**

Delayed wound healing reduces the quality of life (QOL) of patients. Thus, understanding the mechanism of wound healing is indispensable for better management. However, the role of innate immunity in wound healing is thus far unknown. Recently the involvement of TLR3 in wound healing has been evaluated. The systemic administration of polyriboinosinic-polyribocytidylic acid (poly I:C ; a substitute for viral dsRNA and a ligand of toll-like receptor 3), enhances wound healing in vivo. The aim of this study is to improve our understanding of the link between innate immunity and human wound healing, particularly in re-epithelialization.

**Results:**

The present study showed that poly I:C significantly accelerated collective HaCaT cell migration in a scratch assay. Poly I:C also increased IL-8 and bFGF production, and anti-IL-8 antibodies significantly inhibited the migration caused by poly I:C. Human recombinant IL-8 also accelerated collective HaCaT cell migration. An immunofluorescence assay and enzyme-linked immunosorbent assay (ELISA) also revealed that poly I:C decreased E-cadherin protein levels and increased vimentin protein levels, and anti-IL-8 antibody reversed this effect. In contrast, nucleic/cytosolic protein ratios of Snail 1 were unchanged in all tested conditions.

**Conclusion:**

Our findings demonstrated that poly I:C accelerated collective HaCaT cell migration via autocrine/paracrine secretions of IL-8 and the subsequent incomplete epithelial-mesenchymal transition (EMT). Our findings provide a new strategy for wound healing by regulating innate immune systems in re-epithelialization.

## Background

The skin is the frontline of innate immunity and works as a physical and chemical barrier. Thus, skin injury enables the entry of pathological microorganisms and disrupts homeostatic function. Though a skin wound is usually repaired rapidly, wound healing is delayed in the elderly and/or in patients with diabetes mellitus. Delayed wound healing increases the risk of morbidity in diabetic foot ulcers [1] and pressure ulcers [2]. Thus, understanding the mechanism underlying wound healing is needed to develop better treatments. Recently, accumulating data have indicated important roles of innate immunity in wound healing [3]. Stakeholders in innate immunity include toll-like receptors (TLRs), which recognize pathogen-associated molecular patterns (PAMPs) from invading pathogens and damage-associated molecular patterns (DAMPs) released from injured tissues and cells [4]. Therefore, the recognition of PAMPs or DAMPs via TLRs triggers an inflammatory response in both sterile and non-sterile conditions during wound healing [5].

TLRs have negative and positive roles in wound healing. In diabetic foot ulcers, the recognition of DAMPs by TLRs has been proposed to lead to an excessive and prolonged inflammatory response, resulting in impaired wound healing [6]. However, the beneficial effects of TLRs in wound healing have also been reported. For example, TLR4 plays an essential role in early skin wound healing [7]. HMGB1, an endogenous ligand of TLR4, accelerates wound healing [8, 9], whereas a bacterial lipopolysaccharide, an exogenous ligand of TLR4, delayed cutaneous wound healing [10]. Activation of TLR9 by CpG oligodeoxynucleotides accelerates wound healing [11]. Deficiency in Nod2, a cytoplasmic recognition receptor for multiple host patterns, also results in delayed wound healing [12]. In addition to studies on TLR4 and TLR9, the involvement of TLR3 in wound healing has been recently evaluated. The systemic administration of polyriboinosinic-polyribocytidylic acid (poly I:C), a ligand of TLR3, enhances wound healing in vivo [13]. In recent studies, skin wound repair was significantly delayed in TLR3 null mice [14], and poly I:C promoted wound repair of human and murine skin [15]. Increases in the expression of genes involved in skin barrier formation, lipid accumulation, and epidermal organelles were observed with poly I:C stimulation [16]. Interestingly, TLR3 signaling is involved in hair neogenesis after wound formation [17]. Thus, it is important to understand the correlation between wound healing and the skin virome or DAMPs, which could in turn lead to better treatments. However, the roles of viral flora in non-pathological conditions and the involvement of innate immunity remain unclear. Here we investigated the effects of poly I:C on the collective migration of an immortalized human keratinocyte cell line (HaCaT cells). The migration and proliferation of keratinocytes were observed beginning in the intermediate phase of inflammation [18]. This process, known as epithelialization, is a crucial component of wound repair, sealing the epidermal defect and re-establishing barrier function [19, 20]. The aim of this study was to improve our understanding of the link between innate immunity and human wound healing, particularly in re-epithelialization.

## Methods

### Cell culture

HaCaT cells were kindly provided by Dr. N.E. Fusenig (German Cancer Research Center, Heidelberg, Germany) and grown in Dulbecco’s Modified Eagles Medium (DMEM) (Gibco, Carlsbad, CA, USA) containing 10% fetal bovine serum, 100 U/ml penicillin and 100 U/ml streptomycin at 37 °C in 5% humidified CO_2_.

### Scratch assay

HaCaT cells were grown to confluence on 24-well microplates (Iwaki Glass, Chiba, Japan). A linear scratch was made using a 2 mm-wide Cell Scratcher™ (Iwaki Glass), and the wells were washed once with phosphate buffered saline (PBS). Immediately after washing, 0.01, 0.1, or 1 μg/ml poly I:C (Sigma, St. Louis, MO, USA) was added to the cultures. To inhibit poly I:C stimulation, 30 μg/ml chloroquine diphosphate (Wako Pure Chemical Industries, Osaka, Japan), dissolved in the same culture medium, was added. Cells were then fixed and stained with 7.5% formaldehyde and 0.25% crystal violet (Wako Pure Chemical Industries) at 0, 24, 48, or 72 h after the scratch. Grid seals (Iwaki Glass) were affixed onto the bottoms of the wells, and images of the remaining wound area in a 3 × 8 mm^2^ rectangle, at the center of the well (approximately one-half of the scratch wound), were obtained under a stereoscopic microscope and measured using Image J software (NIH). To inhibit proliferation, cells were treated with 10 μg/ml of mitomycin C (Kyowa Hakko Kirin Co, Tokyo, Japan) for 2 h and then washed with PBS once immediately before the scratch was made. To prevent the confounding effect of IL-8, anti-IL-8 antibody (1:200; IBL, Gunma, Japan) was added to the medium immediately after the scratch. Human recombinant IL-8 (50 or 500 ng/ml; Sigma) was also added just after the scratch to confirm the effects of IL-8.

### Cell viability assay

HaCaT cells were seeded (5000 cells per well) onto a 96-well microplate (Iwaki Glass, Chiba, Japan) 24 h before poly I:C, and chloroquine were added at the same concentrations as described above. Next, the cells were incubated for 24, 48, or 72 h, and cell viability was measured using a Cell Counting Kit-8 (Dojindo Laboratories, Kumamoto, Japan) according to the manufacturer’s instructions. The cell density in each well was measured at 450 nm using a microplate reader (iMark Microplate Absorbance Reader, Bio-Rad, Hercules, CA, USA).

### Enzyme-linked immunosorbent assay (ELISA) of IL-8, transforming growth factor (TGF)-β1, E-cadherin, vimentin, Snail, and basic fibroblast growth factor (bFGF)

The IL-8, TGF-β1 and bFGF concentrations in culture medium were measured using an ELISA kit (R&D Systems, Minneapolis, MN, USA) according to the manufacturer’s instructions. The culture supernatants with or without poly I:C and anti-IL-8 antibody at the same doses as described above were collected at 24 h after the scratch and centrifuged at 3000 rpm for 15 min. Cell-free supernatants were then harvested and stored at −20 °C (for IL-8) or −80 °C (for TGF-β1 and bFGF) until further assay. The concentrations in each well were measured at 450 nm using a microplate reader (Bio-Rad). We also measured the protein levels using an ELISA kit of E-cadherin (R&D Systems), Vimentin (Cell Signaling Technology, Danvers, MA, USA) and Snail (Cloud-Clone Corp, Houston, TX, USA) according to the manufacturer’s instructions. Whole cell lysates were extracted using cell lysis buffer (Cell Signaling Technology) or nucleic/cytosolic fractions were extracted using cell fractionation kit-standard (Abcam) according to the manufacturer’s instructions and stored at −20 °C prior to use. Protein concentrations were determined using the RC DC protein assay kit (Bio-Rad).

### Immunofluorescence assay

HaCaT cells were grown to confluence on 24-well microplates (Iwaki) and scratched with or without the combination of 0.1 μg/ml poly I:C and anti-IL-8 antibody (1:200) as described above. Cells were fixed with 4% paraformaldehyde 24 h after the scratch. Cells were washed with 1% bovine serum albumin (BSA, Sigma) in PBS and incubated with 3% BSA for 30 min. Next, the cells were incubated with anti-IL-8 antibody (1:50; IBL), anti-TGF-β1 antibody (1:100; Cell Signaling Technology), anti-E-cadherin antibody (1:100; Abcam, Cambridge, UK), anti-vimentin antibody (1:150; Abcam), and anti-Snail 1 antibody (1:150; Abcam) for two hours at room temperature. Then, the cells were washed and incubated with the secondary antibody, CF488A-labeled anti-rabbit antibody (Biotium, Hayward, CA, USA), for 30 min at room temperature. For negative controls, the primary antibody was replaced with 1% BSA in PBS. Samples were counterstained with 4′,6-Diamidino-2-Phenylindole, Dihydrochloride (Biotium) for nuclear staining. To maintain cytokines in the cells, brefeldin A (50 μg/ml; Sigma) was added to the culture medium 4.5 h before fixation (only for IL-8 and TGF-β1 staining).

### Bright field imaging of the scratched edge margin

HaCaT cells were grown to confluence on 24-well microplates (Iwaki) and scratched with or without 0.1 μg/ml poly I:C. After 24 h of incubation, bright field images were obtained using EVOS FLoid Cell Imaging Station (Thermo Fisher Scientific, Boston, Massachusetts, USA).

### Statistical analysis

Statistical comparisons were performed using the Tukey-Kramer test or Mann–Whitney test. Data are presented as the mean ± SEM. A probability value of < 0.05 was considered to represent a significant difference.

## Results

### Effects of poly I:C on the collective migration of HaCaT cells

**Fig. 1:**
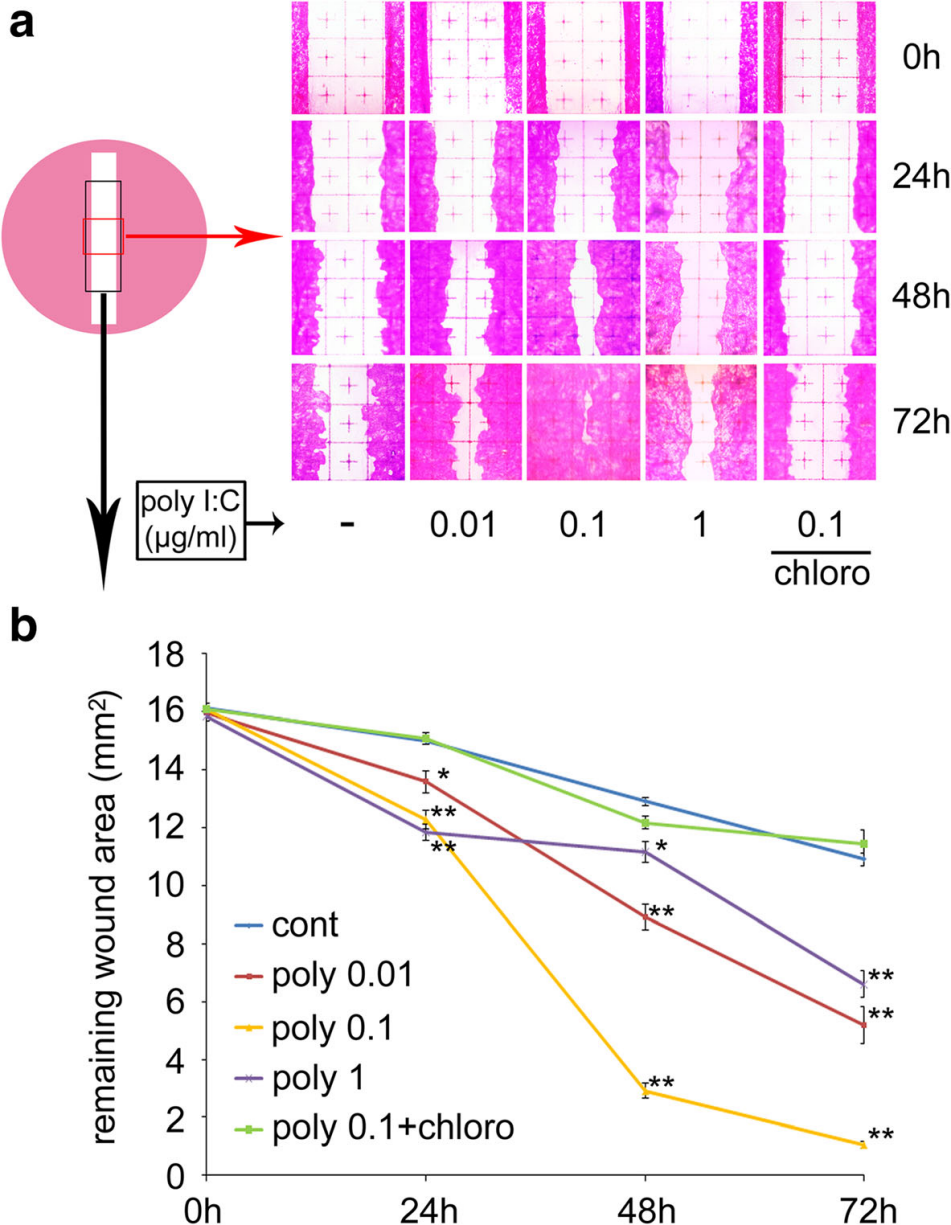
The effects of poly I:C on the scratch assay with HaCaT cells. **a** Representative photographs showing the HaCaT cell remaining wound area at 24, 48, and 72 h after the scratch. **b** The remaining wound area in a 3 × 8 mm^2^ rectangle (*n* = 3). Data are presented as the mean ± SEM. *p < 0.05 vs. control, ** *p* < 0.01 vs. control according to the Tukey-Kramer test. Grids = 1 × 1 mm^2^. Chloro; Chloroquine

**Fig. 2:**
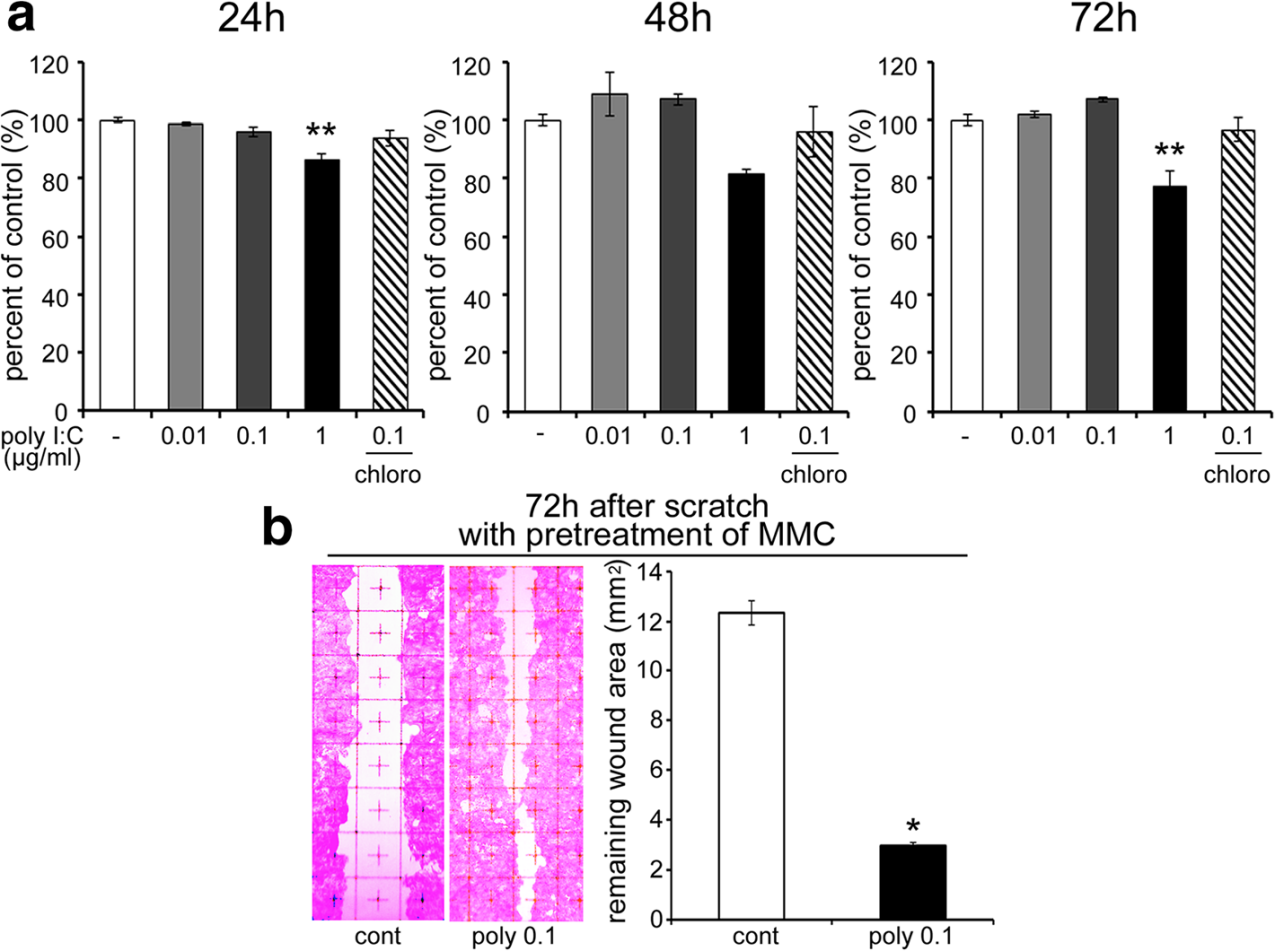
The effects of poly I:C on HaCaT cell viability and collective migration. **a** Cell viability measured by formazan formation at 24, 48, and 72 h after the administration of poly I:C (*n* = 5). **b** Representative images showing collective HaCaT cell migration at 72 h after the scratch with mitomycin C pretreatment for 2 h, and remaining wound area in a 3 × 8 mm^2^ rectangle (*n* = 3). Data are presented as the mean ± SEM. Grids = 1 × 1 mm^2^. *p < 0.05 vs. control according to the Mann–Whitney test. ** *p* < 0.01 vs. control according to the Tukey-Kramer test. poly; poly I:C, Chloro; chloroquine. MMC; mitomycin C

HaCaT cells were exposed to 0.01, 0.1, or 1 μg/ml of poly I:C and subjected to a scratch assay (Fig. 1a, b). Poly I:C significantly decreased the remaining wound area in a dose-dependent manner, which was reversed by chloroquine, a poly I:C inhibitor, at 24, 48, and 72 h after the scratch. Chloroquine alone did not affect the migration (data not shown). However, the remaining wound area was greater at the dose of 1 μg/ml of poly I:C, than at 0.1 μg/ml. Next, we examined the effect of poly I:C on cell viability using a cell counting assay (Fig. 2a). At the doses of 0.01 and 0.1 μg/ml, there were no decreases in cell viability. However, 1 μg/ml of poly I:C decreased the viability, which was significant for 24 and 72 h incubation treatments. The combination of poly I:C (0.1 μg/ml) and chloroquine did not have a significant effect. We also examined the effect of poly I:C on collective migration (Fig. 2b). Cell proliferation was inhibited by pretreatment with mitomycin C (10 μg/ml) for 2 h immediately before scratching, and the remaining wound area was significantly decreased with 0.1 μg/ml of poly I:C 72 h after scratching.

### Involvement of IL-8 in poly I:C-mediated effects

**Fig. 3:**
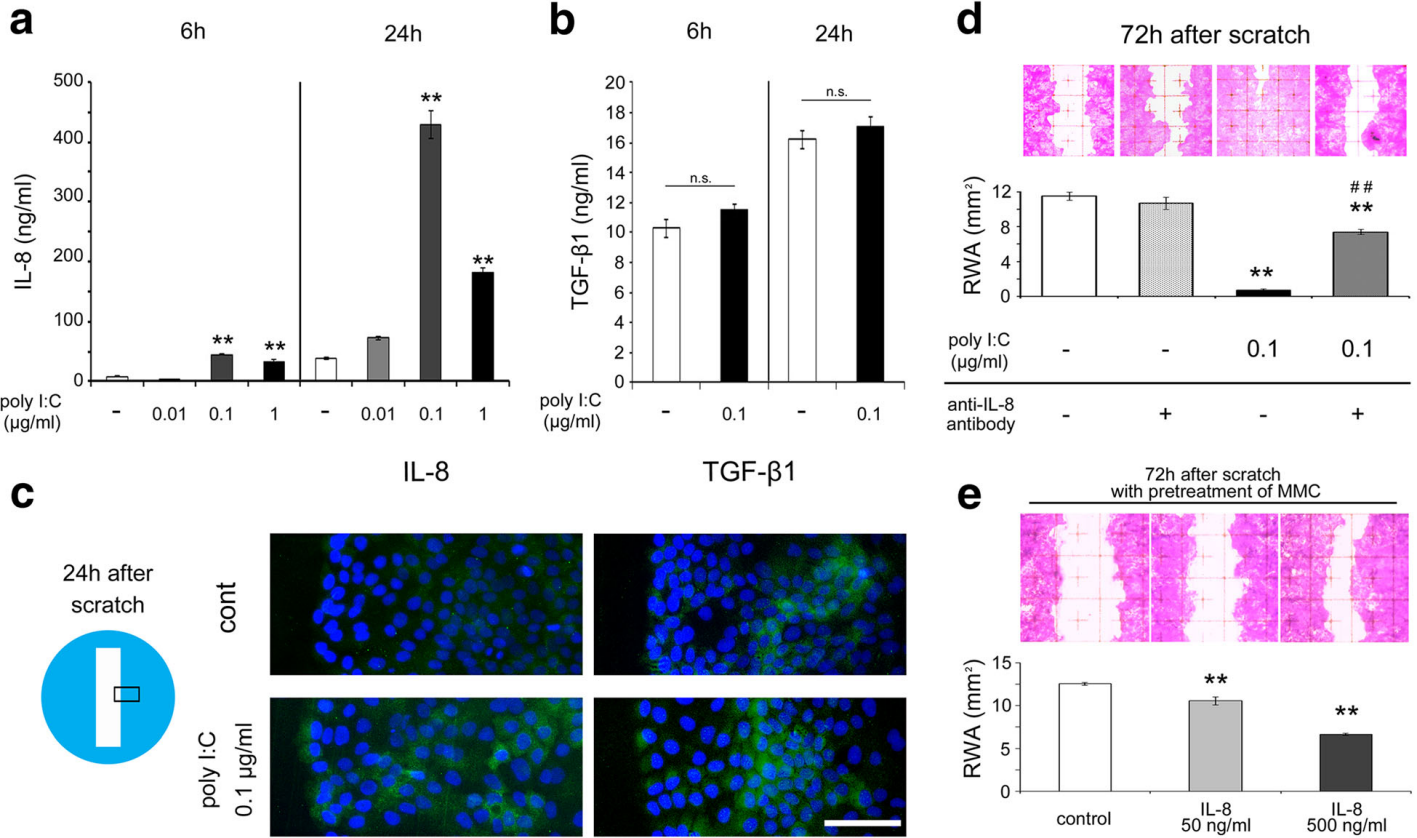
The protein levels and immunohistochemistry results of IL-8 and TGF-β1 and the involvement of IL-8 in migration. **a** The IL-8 protein concentrations after 6 or 24 h of incubation in the culture medium following the scratch and treatment with 0.1 μg/ml of poly I:C (*n* = 3). **b** The TGF-β1 protein concentrations after 6 or 24 h of incubation in the culture medium following the scratch and treatment with 0.1 μg/ml of poly I:C (*n* = 3). **c** Representative immunoreactivity of IL-8 (*green*) or TGF-β1 (*green*) on the scratched edge margins with or without 0.1 μg/ml of poly I:C stimulation at 24 h after the scratch (*Blue*; DAPI). **d** Representative images showing the HaCaT cell remaining wound area at 72 h after the scratch with anti-IL-8 antibody and the remaining wound area in a 3 × 8 mm^2^ rectangle (*n* = 3). **e** Representative images showing the HaCaT cell migration at 72 h after the scratch with human recombinant IL-8 and the remaining wound area in a 3 × 8 mm^2^ rectangle (*n* = 3). Data are presented as the mean ± SEM. * *p* < 0.05 vs. control, ** *p* < 0.01 vs. control, ^##^ *p* < 0.01 vs. poly I:C alone according to the Tukey-Kramer test (**a**, **d**) or the Mann–Whitney test (**b**). Data for the immunofluorescence assays are representative of at least three independent experiments. Grids = 1 × 1 mm^2^. Scale bar: 100 μm. RWA; remaining wound area. MMC; mitomycin C

Some cytokines, such as IL-8 and TGF-β1, are known to induce keratinocyte migration. Poly I:C induced the production of IL-8 from HaCaT cells within 6 and 24 h of incubation after the scratch (Fig. 3a). We also examined the protein levels of TGF-β1. However, poly I:C did not have a significant effect in this condition (Fig. 3b). Immunofluorescence assays of IL-8 and TGF-β1 were performed to evaluate the localization of cytokine production (Fig. 3c). Poly I:C increased the immunoreactivity of IL-8 in a diffuse pattern at 24 h after the scratch. Immunoreactivity of TGF-β1 was also diffuse and not obviously different between control and poly I:C-treated cells. These results were consistent with the ELISA results. Next, we examined whether IL-8 was involved in the poly I:C-mediated effects. Remaining wound areas at 72 h after scratching were evaluated with or without anti-IL-8 antibody (Fig. 3d). Anti-IL-8 antibody alone did not affect the remaining area compared with the control, while the antibody significantly inhibited the effect of poly I:C. Human recombinant IL-8 also increased the migration in a dose-dependent manner (Fig. 3e).

### ELISA and immunofluorescence assays of epithelial-mesenchymal transition (EMT)-related proteins

**Fig. 4:**
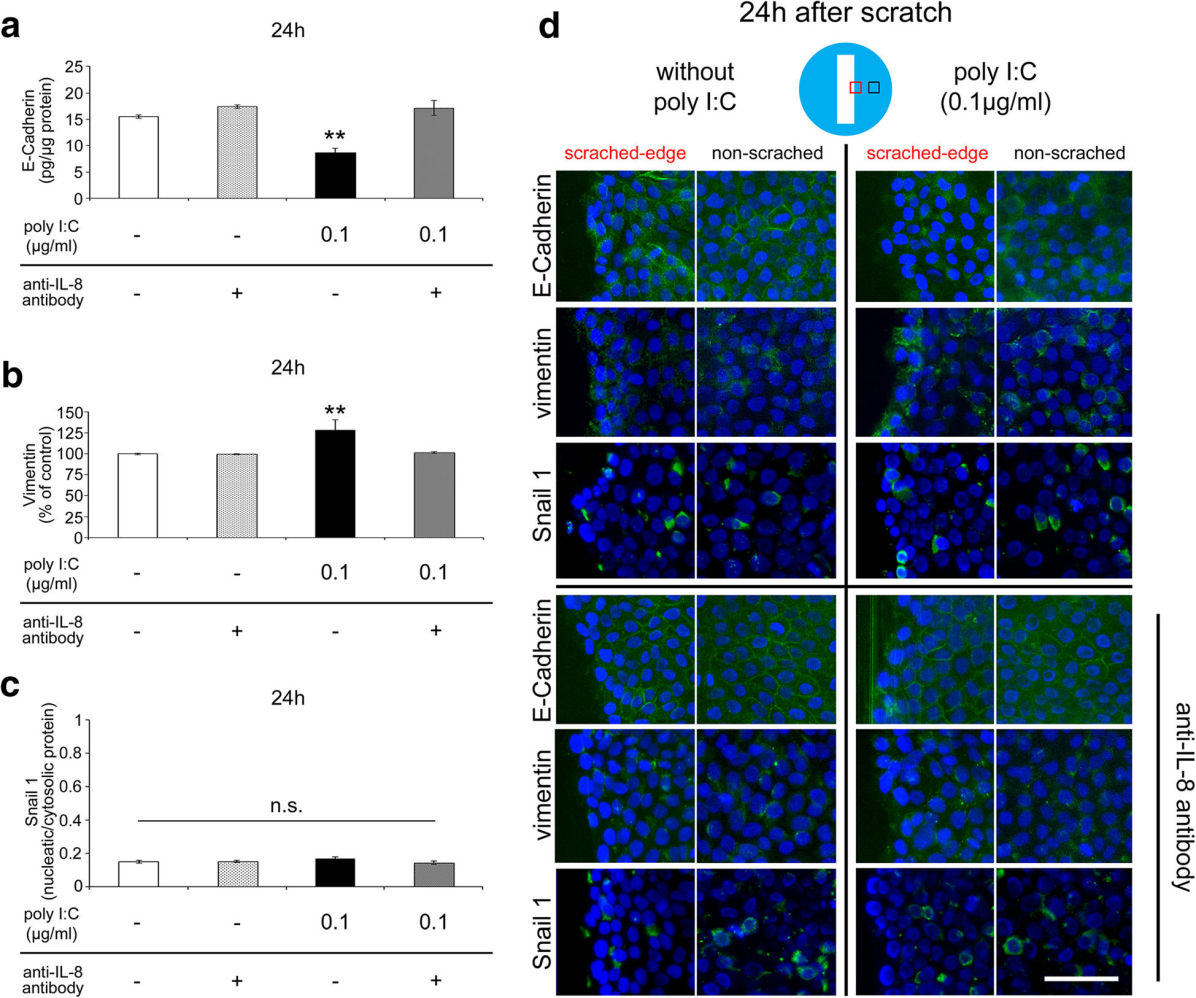
EMT-associated cellular marker alterations. **a** E-Cadherin (epithelial cell marker) protein levels at 24 h after scratching (*n* = 4). **b** Vimentin (mesenchymal cell marker) protein levels at 24 h after scratching (*n* = 4). **c** The nucleus/cytosol ratio of Snail 1 (EMT-related transcriptional factor) protein levels at 24 h after scratching (*n* = 4). **d** Immunoreactivities of E-cadherin (*green*), vimentin (*green*), and Snail 1 (*green*) at 24 h after the scratch (*Blue*; DAPI) 24 h after poly I:C stimulation with or without anti-IL-8 antibody. Data are presented as the mean ± SEM. n.s.: not significant according to the Tukey-Kramer test. Data for immunofluorescence assay are representative of at least three independent experiments. Scale bar: 100 μm

Next, we examined the potential of EMT using ELISA and an immunofluorescence assay at 24 h after the scratch. The protein levels of E-cadherin were significantly decreased only in the poly I:C-treated group (Fig. 4a), and the levels of vimentin were increased in the same group (Fig. 4b). In contrast, the protein levels of Snail 1 in both the nucleus and cytosol were unchanged, and the nucleus/cytosol protein ratios were also unchanged (Fig. 4c). Next, immunofluorescence assays were performed to detect the distributions of these proteins (Fig. 4d). In control cells, positive staining of E-cadherin, an epithelial cell marker, was observed in a pericellular pattern, while the immunoreactivity of vimentin, a mesenchymal cell marker, was weakly detected. The immunoreactivity of Snail was detected mainly in the cytosol and weakly in the nucleus. In contrast, in poly I:C (0.1 μg/ml)-treated cells, E-cadherin immunoreactivity was decreased, and the pericellular pattern was diminished, while the immunoreactivity of vimentin was increased specifically at the scratched edge. The reactivity of Snail was similar to that of the control. These alterations were not observed in the presence of anti-IL-8 antibody.

### Category of collective migration

**Fig. 5:**
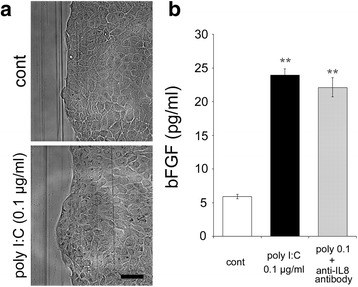
Bright field image of migration and protein levels of bFGF. **a** Representative bright field images on the scratched edge margins with or without 0.1 μg/ml of poly I:C stimulation at 24 h after the scratch. **b** The bFGF protein concentrations after 24 h of incubation in the culture medium following the scratch and treatment with 0.1 μg/ml of poly I:C with or without anti-IL-8 antibody (*n* = 3). Data of bright field images are representative of at least three independent experiments. Data are presented as the mean ± SEM. ** *p* < 0.01 vs. control according to the Tukey-Kramer test. Scale bar: 100 μm

The effect of the mitomycin C treatment on migration (Fig. 2b) indicated that the effect of poly I:C did not depend on cell proliferation. Thus, we examined collective migration. Bright field images in both control and poly I:C-treated samples showed a “sheet migration”-like phenomena (Fig. 5a), and the protein levels of bFGF, a stimulant of sheet migration, were significantly increased by poly I:C (0.1 μg/ml) stimulation in culture medium after 24 h of incubation. This effect was not reversed by the anti-IL-8 antibody (Fig. 5b).

## Discussion

Various mechanisms are involved in the TLR3-related wound healing process. In TLR3 null mice, insufficient recruitment of neutrophils and macrophages was observed, and the expression of chemokines was also decreased in wounds [14]. In addition, poly I:C enhanced the accumulation of leukocytes and upregulated chemokine expression in wound healing [15]. TLR3 was also required for skin barrier repair [21]. To investigate the effects of poly I:C in wound healing, we focused on keratinocytes. We demonstrated that poly I:C accelerated the migration of HaCaT cells. Moreover, poly I:C induced IL-8 without affecting TGF-β1 secretion. Anti-IL-8 antibody inhibited the effect of poly I:C, and the antibody also inhibited poly I:C-evoked EMT-related cellular marker alterations. Poly I:C also increased the secretion of bFGF. Taken together, these results suggested that IL-8 produced by keratinocytes via TLR3 stimulation affects wound healing.

Interestingly, poly I:C appears to have opposing actions in wound healing. Although 0.01 and 0.1 μg/ml of poly I:C did not have cytotoxic effects, keratinocyte death is reportedly caused by excessive poly I:C stimulation [22]. Indeed, our present study revealed that at a higher dose of poly I:C, 1 μg/ml, cell viability was decreased, and the remaining wound area was greater than that at 0.1 μg/ml, although it was still smaller than that observed in the control. Taken together, these data suggest that excessive poly I:C stimulation may delay wound healing.

The potential sources of TLR3 ligands in wound healing should also be discussed. Poly I:C is a ligand of TLR3 and a substitute for both PAMPs of the double-stranded (ds) RNA virus and endogenously generated DAMPs [23, 24]. Although, to the best of our knowledge, there is no evidence supporting the existence of a skin biome of dsRNA viruses, DNA viruses were identified in the skin of healthy humans [25]. It is well known that TLR3 can recognize RNA from any microorganism or dying cell, as mRNA [26] and noncoding RNA [27] are recognized by TLR3. Functional TLR3 is expressed in keratinocytes [28]. Via TLR3 signaling, NF-κB is activated [29], while NF-κB inhibition delays wound healing [30]. Taken together, these studies suggest the important roles of TLR3 in regulating inflammation as a component of wound healing. Although potential sources of the TLR3 ligand in skin wound healing are still unknown, our present observation suggests that TLR3 plays a physiological role in skin wound healing via accelerated keratinocyte migration. Moreover, commensal bacteria appear to play a regulatory role in reducing the TLR3-dependent inflammatory response after skin injury [31]. Thus, the interactions between the skin microbiota, including bacterial and viral residents as well as DAMPs, remain to be elucidated.

After stimulation of TLRs, many types of cytokines are induced and play differential roles in wound healing. The role of IL-8 in wound healing appears to be complicated. In vivo, topical IL-8 administration significantly diminishes wound constriction [32], whereas, in humans, the level of IL-8 from an unhealed wound biopsy is significantly higher than that of normal skin [33]. The most abundant cytokines in wound fluid reported in surgical drains are IL-6 and IL-8 [34]. This finding might be the result of excessive inflammation caused by IL-8-mediated neutrophil recruitment [35] or it may be a representation of keratinocyte death, as poly I:C-induced cytotoxicity and IL-8 secretion have a parallel dose dependence [36]. In the present study, increased secretion of IL-8 by poly I:C was detected by ELISA and recombinant IL-8 increased collective cell migration. These observations were consistent with those of previous studies reporting that poly I:C induces IL-8 in HaCaT and human epidermal keratinocytes [37, 38] and that IL-8 increases HaCaT cell migration [39, 40]. In null CXCR2 mice, which lack a functional IL-8 receptor, wound healing is delayed [41, 42], and in human skin, topical administration of IL-8 increased the length of the re-epithelialized area [32]. These data indicated the advantageous nature of IL-8 in re-epithelialization during wound healing and show that poly I:C may enhance the autocrine positive-feedback loop of chemokines in epithelialization [43]. In this study, positive correlations were observed between the concentration of IL-8 at the dose of 0.1 and 1 μg/ml and the results of scratch assays. However, at the dose of 0.01 μg/ml, the concentration was not significantly increased, although the remaining wound area was significantly smaller than that of control at 24 h after scratching. Taken together, these results suggest that IL-8 was expended in the culture medium by autocrines. Next, we demonstrated that anti-IL-8 antibody showed a limited inhibitory effect (approximately 70% of the control), suggesting that other mechanisms (i.e., cytokines) are involved in poly I:C-induced collective migration. Further studies to identify other factors that induce the migration caused by poly I:C are needed. In addition, all results presented here were obtained from in vitro studies. Although the scratch assay is a simple, versatile, and cost-effective method, it has some limitations [44]. Thus, more complex methods, such as 3D cell culture systems and in vivo studies, are required to confirm our results.

Unlike the results obtained for IL-8, an increase in TGF-β1 was not observed in the present study. TGF-β1 promotes keratinocyte migration [45], and systemic administration of poly I:C increases the mRNA levels of TGF-β1 in excised wounds [46]. At the protein level, TGF-β1 production in poly I:C-stimulated human aortic valve interstitial cells increases in a dose-dependent manner [47]. These data suggest that TGF-β1 may be produced in greater quantities by cells other than keratinocytes as a part of wound healing [48]. The interactions between TGF-β1 and innate immunity may be important during wound healing.

In addition to the cytokines, cumulative studies have shown the importance of EMT in cell migration. In EMT, cells lose “cell-to-cell” adhesion, have reduced basal cell polarity, acquire a fibroblastic phenotype, and have increased cell motility to migrate or metastasize [49], although EMT can be reversible and is often an incomplete process [50]. In human biliary epithelial cells, poly I:C induces EMT, while the expression levels of TGF-β1 and vimentin are not affected [51]. In the present study, the TGF-β1 concentration was not affected by poly I:C alone; however, the immunoreactivity of vimentin was increased. This inconsistency may have been caused by the difference in cell types.

Recent studies have also shown that collective migration is sometimes not accompanied by complete EMT but rather by incomplete EMT, although complete EMT generally causes single cell migration [52]. In this study, approximately half the amount of E-cadherin protein was still observed, and we did not observe significant translocation of Snail 1 from the cytoplasm to the nucleus 24 h after poly I:C stimulation. The unaltered state of Snail 1 may be explained by the present observation that an increase in TGF-β1 was not observed because TGF-β induces the expression of Snail 1 [49]. These findings indicate that incomplete EMT may be induced by a variety of RNAs during wound healing.

Thus far, little is known regarding the identity of the factor that determines complete or incomplete EMT [53]. In the present study, anti-IL-8 antibody inhibited EMT-related cellular marker alterations, indicating that IL-8 may be involved in this process. In tumor cells, IL-8 also induces EMT [54]. Tumor collapse could result in the release of RNA, which may evoke EMT via TLR3, resulting in greater invasion or metastasis by the self-secretion of IL-8 from the tumor. In breast cancer, tumors with a high expression of TLR3 were associated with a significantly greater probability of metastasis [55]. In other hands, with the lack of constitutive activation of RAS signaling pathways, TGF-β1 induced an incomplete and transient mesenchymal conversion [56]. Epithelial growth factor (EGF) also induced incomplete EMT in squamous cell carcinoma [57]. Further studies regarding the roles of cytokine and innate immunity in the completeness of EMT in wound healing are needed.

We finally examined the categories of collective migration. According to the results of the bright field images, less sprouting, branching, and slug-like behavior were observed [50]. In addition, bFGF, an important stimulant of sheet migration [58], was significantly increased. These data suggests that the collective migration induced by poly I:C may be bFGF- mediated sheet migration. However, the bFGF production was not inhibited by the anti-IL-8 antibody, indicating the independence of IL-8 in bFGF secretion.

## Conclusions

In conclusion, our findings demonstrated that poly I:C accelerated collective HaCaT cell migration via autocrine/paracrine secretions of IL-8 and subsequent incomplete EMT. The present study helps improve our understanding of the important role of innate immunity in human wound healing.

## Abbreviations

bFGF: Basic fibroblast growth factor
DAMPs: Damage-associated molecular patterns
EMT: Epithelial-mesenchymal transition
IL: Interleukin
PAMPs: Pathogen-associated molecular patterns
poly I:C: Polyriboinosinic-polyribocytidylic acid
TGF: Transforming growth factor
TLRs: Toll-like receptors
